# Niacin in Pharmacological Doses Alters MicroRNA Expression in Skeletal Muscle of Obese Zucker Rats

**DOI:** 10.1371/journal.pone.0098313

**Published:** 2014-05-21

**Authors:** Aline Couturier, Janine Keller, Erika Most, Robert Ringseis, Klaus Eder

**Affiliations:** Institute of Animal Nutrition and Nutrition Physiology, Justus-Liebig-Universität Giessen, Giessen, Germany; Ospedale Pediatrico Bambino Gesu', Italy

## Abstract

Administration of pharmacological niacin doses was recently reported to have pronounced effects on skeletal muscle gene expression and phenotype in obese Zucker rats, with the molecular mechanisms underlying the alteration of gene expression being completely unknown. Since miRNAs have been shown to play a critical role for gene expression through inducing miRNA-mRNA interactions which results in the degradation of specific mRNAs or the repression of protein translation, we herein aimed to investigate the influence of niacin at pharmacological doses on the miRNA expression profile in skeletal muscle of obese Zucker rats fed either a control diet with 30 mg supplemented niacin/kg diet or a high-niacin diet with 780 mg supplemented niacin/kg diet for 4 wk. miRNA microarray analysis revealed that 42 out of a total of 259 miRNAs were differentially expressed (adjusted P-value <0.05), 20 being down-regulated and 22 being up-regulated, between the niacin group and the control group. Using a biostatistics approach, we could demonstrate that the most strongly up-regulated (log2 ratio ≥0.5) and down-regulated (log2 ratio ≤−0.5) miRNAs target approximately 1,800 mRNAs. Gene-term enrichment analysis showed that many of the predicted target mRNAs from the most strongly regulated miRNAs were involved in molecular processes dealing with gene transcription such as DNA binding, transcription regulator activity, transcription factor binding and in important regulatory pathways such as Wnt signaling and MAPK signaling. In conclusion, the present study shows for the first time that pharmacological niacin doses alter the expression of miRNAs in skeletal muscle of obese Zucker rats and that the niacin-regulated miRNAs target a large set of genes and pathways which are involved in gene regulatory activity indicating that at least some of the recently reported effects of niacin on skeletal muscle gene expression and phenotype in obese Zucker rats are mediated through miRNA-mRNA interactions.

## Introduction

Niacin (nicotinic acid) is a water-soluble vitamin of the B-complex involved in many different metabolic reactions as a precursor of the coenzymes nicotinamide adenine dinucleotide (NAD) and nicotinamide adenine dinucleotide phosphate (NADP) [1]. At pharmacological doses (2–6 g/d), niacin has long been used for the clinical therapy of different forms of dyslipidemia, particularly hypertriglyceridemia, in humans due to the fact that niacin has potent lipid-modulating activities (lowering of triacylglycerols (TAG), LDL-cholesterol, and lipoprotein (a), increasing HDL-cholesterol) [1]–[5]. Apart from these lipid-modulating effects of niacin, which have been suggested to involve inhibition of lipolysis in adipose tissue [4], and reduction of gene expression of APOC3, which is known to inhibit hydrolysis of VLDL-TAG, in the liver [6], niacin was also recently reported to have pronounced effects on skeletal muscle gene expression and skeletal muscle phenotype in obese Zucker rats [7]. Using this genetic model of obesity, metabolic syndrome and diabetes, we observed that administration of a pharmacological dose of niacin for 4 wk causes a muscle fiber shift from type II (glycolytic) to type I (oxidative) in skeletal muscle [7]. In addition, we observed that the expression of genes involved in fatty acid transport, mitochondrial fatty acid import and oxidation, oxidative phosphorylation and angiogenesis in skeletal muscle is elevated by niacin administration [7], indicating a change of the muscle metabolic phenotype towards a more oxidative one. Moreover, we found that genes encoding molecular regulators of muscle fiber distribution, like peroxisome proliferator-activated receptor δ (PPARδ), PPARγ coactivator-1α (PGC-1α) and PGC-1β, are strongly induced by niacin in skeletal muscle of the obese Zucker rats [7]. Up-regulation of these transcription factors by niacin administration is likely responsible for the muscle fiber switch from type II to type I because PPARδ and PGCs are critical regulators of muscle fiber distribution and muscle metabolic phenotype [8]–[12]. It is currently unknown, however, how pharmacological niacin doses regulate gene expression in skeletal muscle.

MicroRNAs (miRNAs) represent a relatively newly identified class of regulatory molecules which have important functions for gene expression. miRNAs are small (∼19–24 nucleotides) endogenous RNAs, which regulate gene expression mainly at the posttranscriptional level through binding to complementary mRNA sequences leading to degradation of the specific mRNAs or repression of protein translation, and, thus, inhibition of gene expression. Whether the mRNA is degraded or protein translation is repressed depends largely on the degree of miRNA-mRNA sequence complementarity. Perfect sequence complementarity has been shown to result in the cleavage of the mRNA strand, whereas less complementarity leads to the repression of protein translation [13], [14]. Interestingly, a single miRNA can regulate hundreds of protein encoding target mRNAs indicating the great regulatory potential of miRNAs [15], [16]. The significant role of miRNAs for regulating gene expression becomes also evident from estimations that nearly 30% of the human genes represent miRNA targets [16].

Recent studies have already shown that several dietary factors, such as vitamin D, biotin, cholesterol, and conjugated linoleic acids [17]–[23], influence miRNA expression in tissues which indicates that nutrient-gene interactions can be mediated at least partially by miRNA-mRNA interactions. To our knowledge it has not been studied yet whether administration of niacin at pharmacological doses influences miRNA expression. The aim of the present study, therefore, was to investigate the influence of niacin at pharmacological doses on the miRNA expression profile in skeletal muscle of obese Zucker rats. Muscle tissue was used for miRNA profiling in an attempt to explain some of the recently observed effects of niacin on skeletal muscle gene expression in this animal model by an altered expression of miRNAs.

## Materials and Methods

### Animal experiment

The animal experiment was approved by the local Animal Care and Use Committee (Regierungspräsidium Gieβen). As animal model, we used the obese (fa/fa) Zucker rat (Crl:ZUC-*Lepr^fa^*; Charles River, France), which is a widely used genetic model of obesity, metabolic syndrome and diabetes, and, by comparison with the lean Zucker rat, exhibits hyperphagia, hyperinsulinemia, and hyperlipidemia. For this study, we used plasma and muscle samples from obese Zucker rats of a recently performed experiment of our group [7], in which twelve, 8- to 10-wk old male obese Zucker rats were randomly divided in two groups (control group and niacin group, 6 animals/group). The rats were fed two different semi-purified diets composed according to the recommendations of the National Research Council for laboratory rats [24]. The first diet containing 30 mg supplemented niacin per kg diet, which was sufficient to cover the niacin requirement, was fed to the “control” group, whereas the second diet containing 780 mg supplemented niacin (Lonza, Basel, Switzerland) per kg diet was fed to the “niacin” group. Both diets consisted of (g/kg diet): corn starch, 530; casein, 200; saccharose, 100; soybean oil, 70; cellulose, 50; minerals, 30; and vitamins, 20. Niacin was added to the “niacin” diet at the expense of corn starch. The dry components of the diets were mixed and the mixture pressed under high pressure in a pelletizing machine (Typ 14–175, Kahl, Reinbek, Germany) to yield cylindrical pellets. The diets were fed *ad libitum* and water was available *ad libitum* for a period of 28 days. At the end of the experiment the rats were decapitated under CO_2_ anesthesia. Blood was collected and plasma obtained by centrifugation, and skeletal muscle (*M. rectus femoris*) was excised and immediately stored at −80°C. Further details regarding diet composition, animal keeping and sample collection are shown in our previous publication [7]. In Accordance with Article 4 par. 3 of the German Animal Welfare Law all animals were humanely killed for scientific purpose approved by the Animal Welfare Officer of the Justus-Liebig-University, JLU No. 450_AZ.

### Determination of plasma concentrations of nicotinic acid, nicotinamide (NAM), and nicotinuric acid (NUA)

The concentrations of nicotinic acid, NAM and NUA in plasma were determined by LC-MS/MS according to the method from Liu et al. [25] with slight modifications. Frozen samples were thawed at RT, centrifuged at 600×*g* for 2 min, and a 200 µl aliquot was transferred into a 1.5 ml tube. Then, 10 µl each of internal standard solution (Oxiracetam) (in methanol) and methanol/water (5∶95, v/v) was added, the tube gently mixed and after adding 500 µl acetonitrile the mixture was vortexed for 1 min. The mixture was centrifuged at 13,000×*g* for 10 min at 10°C, and 400 µl of the supernatant was transferred to another 1.5 ml tube. Subsequently, the mixture was evaporated to dryness at 40°C under a stream of nitrogen. The residue was reconstituted in 200 µl of methanol/water (10∶90, v/v) acidified with 0.1% formic acid. Finally, the reconstituted solution was centrifugated at 13,000×*g* for 5 min at RT, and an aliquot of the solution was injected into the LC-MS/MS system for the analysis. The HPLC system was a Hitachi LaChromUltra system (Darmstadt, Germany) consisting of the L-2160U solvent delivery modules, an L-2200U autosampler, an L-2300 column oven, and a system controller. Chromatography was performed on a Nucleodur Polartec analytical column (particle size 3 µm, length 150 mm, internal diameter 3 mm; Macherey-Nagel, Düren, Germany) fitted with a Nucelodur Polartec EC gurad column (particle size 3 µm, 4×3 mm; Macherey-Nagel). The mobile phase consisted of methanol/H_2_O (10∶90, v/v) acidified with 0.1% formic acid. The flow rate was 0.35 ml/min with column temperature maintained at 35°C. For mass analysis and detection an API 3200 triple-quadrupole mass spectrometer (Applied Biosystems/MDS SCIEX, Darmstadt, Germany) equipped with an electrospray ionization (ESI) source. Analyst software (Applied Biosystems/MDS SCIEX, Darmstadt, Germany) was used for data acquisition and processing. The standard curves were found to be linear in the range of 0.08–16.4 µmol/l for nicotinic acid, 2.05–40.9 µmol/l for NAM, and 0.08–16.4 µmol/l for NUA. The mean correlation coefficient for each analyte was 0.999 and the detection limit was 0.08 µmol/l. The precision estimated by analyzing 3 replicates of rat plasma was 3.5% for NAM, whereas no signal could be detected for nicotinic acid and NUA.

### RNA isolation

Total RNA, including small RNAs, was isolated from *M. rectus femoris* using the Qiagen miRNeasy Mini Kit (Qiagen, Hilden, Germany) according to the manufacturer's protocol. The isolated RNA was used for miRNA microarray analysis and subsequent qRT-PCR analysis. Concentration of total RNA was determined using an Infinite 200 M microplate reader and a nanoQuant Plate (both from Tecan, Männedorf, Switzerland) and its integrity was confirmed by 1% agarose gel electrophoresis. Isolated RNA was immediately frozen and stored at −80°C.

### miRNA microarray analysis

The miRNA microarray analysis was performed by Exiqon Services (Denmark). Sample total RNA quality was verified using an Agilent BioAnalyzer 2100 System. MiRCURY LNA microRNA Arrays (7th Gen) following the miRBASE release 18 (http://www.mirbase.org/) were used to analyze the expression profile of each sample (Exiqon, Denmark). 750 ng total RNA from sample and reference was labeled using the mercury LNA microRNA Hi-Power Labeling Kit, Hy3/Hy5 (Exiqon, Denmark) and were mixed pair-wise and hybridized according to the instruction manual using a Tecan HS4800 hybridization station (Tecan, Austria). Afterwards, slides were scanned using the Agilent G2565BA Microarray scanner System (Agilent Technologies, Inc., USA) and image analysis was carried out the ImaGene 9 (mercury LNA microRNA Array Analysis Software, Exiqon, Denmark). The quantified signals were background corrected (Normexp with offset value 10, [27]) and normalized using the global Lowess (LOcally WEighted Scatterplot Smoothing) regression algorithm. The signal values were filtered based on absent/present calls. miRNAs with present calls <20% were removed from the final dataset used for the expression analysis. The microarray data related to all samples have been deposited in NCBI's Gene expression Omnibus public repository [28].

### qRT-PCR validation of differentially expressed miRNAs

For validation of results from microarray analysis, eight of the differentially expressed miRNAs identified by microarray analysis were analyzed by qRT-PCR using a Rotor-Gene 2000 system (Corbett Research, Mortlake, Australia). For this, cDNA was synthesized from 0.5 µg of total RNA using the miScript II RT Kit (Qiagen, Hilden, Germany) according the manufacturer's protocol and stored at −20°C in 1∶10-diluted aliquots. 2 µl cDNA, 2 µl miScript Universal Primer (10×), 2 µl miScript Primer Assay (10×) and 4 µl RNase free and 10 µl QuantiTect SYBR Green PCR Master Mix (2×) (all from Qiagen, Hilden, Germany) were mixed and qRT-PCR was performed: 15 min incubation at 95°C, 40 cycles of a three-stage temperature profile of 94°C for 15 sec and 55°C for 30 sec. Relative changes were calculated using 2^−ΔCt^ equation [29] using expression level of U6 small nuclear RNA (U6 snRNA) as internal control.

### qRT-PCR validation of mRNA targets of differentially expressed miRNAs

For validation of some of the *in silico*-predicted target genes, we determined mRNA levels of a selected set of target genes predicted from down-regulated and up-regulated miRNAs by means of qRT-PCR. cDNA synthesis and qPCR were performed as recently described in detail [30]. For normalization, the three most stable out of six tested potential reference genes were CANX, RPL13, TOP1. Characteristics of gene-specific primers are shown in **Table S1**.

### *In silico*-target prediction of differentially expressed miRNAs and functional analysis

*In silico*-prediction of targets of differentially expressed miRNAs was carried out using three online free available algorithms, TargetScan release version 6.2 (http://www.targetscan.org/), miRanda and miRDB (http://mirdb.org/miRDB/). Functional analysis of the predicted targets of the most strongly regulated miRNAs was performed by enrichment analysis of Gene Ontology (GO) terms and Kyoto Encyclopedia of Genes and Genomes (KEGG) pathways using the Database for Annotation, Visualization and Integrated Discovery (DAVID) gene annotation tool (http://david.abcc.ncifcfr.gov/).

### Statistical analysis

Data shown are means ± SD. Data were analyzed by Student's t test using Minitab statistical software (Release 13, Minitab Inc., State College, PA, USA). Means were considered significantly different for P<0.05. P-values of microarray data have been corrected for multiple testing by the Benjamini and Hochberg adjustment method. miRNAs with an adjusted P-value <0.05 were considered to be differentially regulated by niacin supplementation.

## Results

### Growth performance

Initial and final body weights as well as daily body weight gains did not differ between the control group (367±11 g; 507±18 g; 4.98±0.56 g; n = 6) and the niacin group (357±15 g; 500±29 g; 5.00±0.69 g; n = 6). In addition, daily feed intake and feed conversion ratio did not differ between the control group (25.6±1.4 g/d; 5.17±0.35 g feed/g body weight gain; n = 6) and the niacin group (26.4±1.9 g/d; 5.32±0.44 g feed/g body weight gain; n = 6).

### Plasma concentrations of nicotinic acid and its metabolites (NAM and NUA)

Rats of the niacin group had higher plasma NAM levels than those of the control group (9.88±1.72 vs. 4.96±1.76 µmol/l; n = 6; P<0.05). Plasma concentrations of nicotinic acid and NUA were below the limit of detection (0.08 µmol/l) in both groups.

### Identification of differentially regulated miRNAs in skeletal muscle by miRNA expression profiling

**Figure 1:**
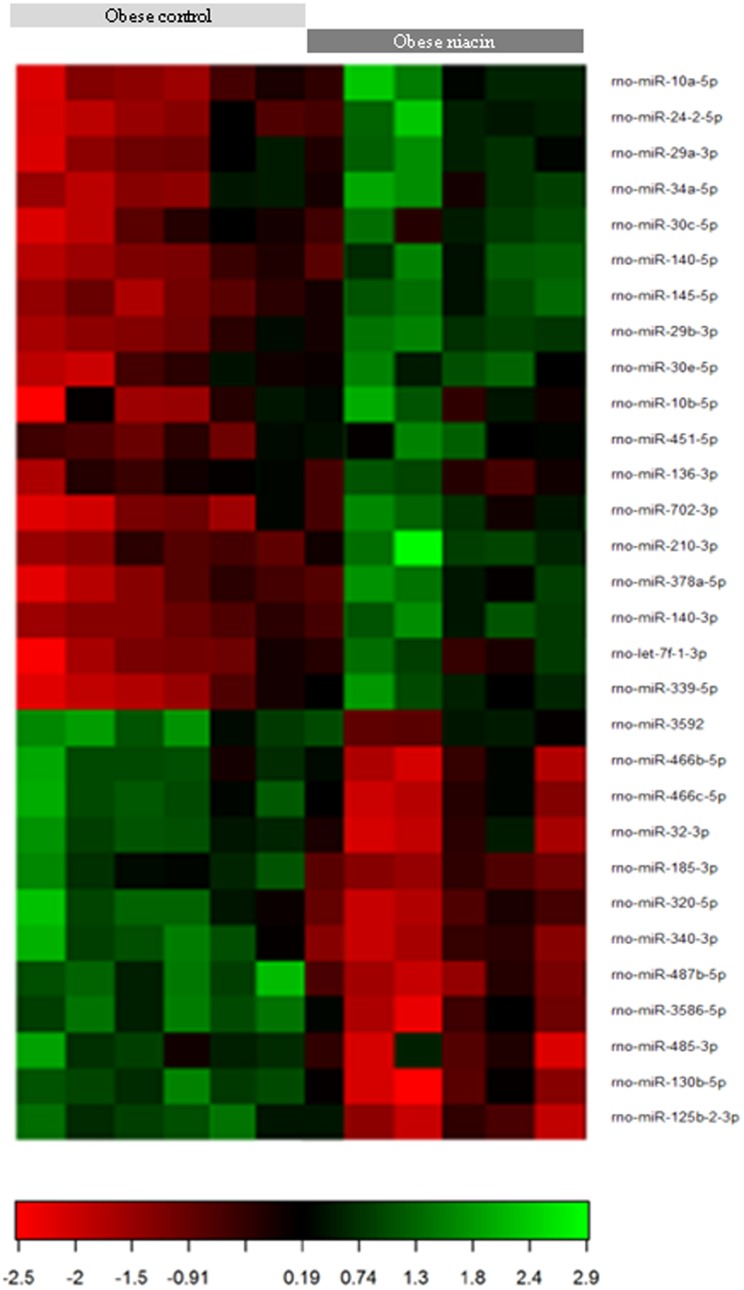
Heat map of the differentially expressed miRNAs in *M. rectus femoris* of obese Zucker rats fed either a control diet with 30 mg supplemented niacin/kg diet (control group) or a high-niacin diet with 780 mg supplemented niacin/kg diet (niacin group) for 4 wk. Each row represents an individual microRNA and each column represents a sample. The color scale illustrates the relative expression level of miRNAs. Red color represents an expression level below the reference channel, and green color represents expression higher than the reference. The codes on the legend are log2-transformed values. Differentially expressed miRNAs chosen with an adjusted P-value <0.05.

miRNA microarray analysis (miCURY LNA microRNA Array 7^th^ Gen) was performed to identify miRNAs regulated by niacin in *M. rectus femoris* of obese Zucker rats. The analysis showed that 42 out of a total of 259 miRNAs were differentially expressed (adjusted P-value <0.05) between the niacin group and the control group. Among the 42 differentially expressed miRNAs, 22 miRNAs were down-regulated and 20 miRNAs were up-regulated in the niacin group compared to the control group. **Figure 1** shows the group-specific signal intensities and the associated log2 ratios and fold changes are given in **Table S2**. The miRNAs with a log2 ratio ≥0.5 and a log2 ratio ≤−0.5 were designated as significantly up- and down-regulated, respectively, by niacin. According to this filter, 5 miRNAs were significantly up-regulated and 12 miRNAs were significantly down-regulated by niacin (**Table 1**).

**Table 1:** Most strongly up-regulated (log2 ratio ≥0.5) and down-regulated (log2 ratio ≤−0.5) miRNAs in *M. rectus femoris* of obese Zucker rats fed either a control diet with 30 mg supplemented niacin/kg diet (control group) or a high-niacin diet with 780 mg supplemented niacin/kg diet (niacin group) for 4 wk.

Probe ID	Annotation	Log2 ratio	FC	Adj. P-value*
*Up-regulated miRNAs*				
168586	rno-miR-34a-5p	0.545	1.459	0.036
11040	rno-miR-29b-3p	0.538	1.452	0.021
42641	rno-miR-145-5p	0.531	1.445	0.021
42950	rno-miR-24-2-5p	0.524	1.438	0.270
13485	rno-miR-10a-5p	0.511	1.425	0.261
*Down-regulated miRNAs*				
11221	rno-miR-300-3p	−0.507	−1.422	0.312
42694	rno-miR-485-3p	−0.525	−1.439	0.360
42933	rno-miR-466b-5p	−0.533	−1.447	0.301
42586	rno-miR-466c-5p	−0.640	−1.558	0.205
148530	rno-miR-466c-3p	−0.658	−1.578	0.312
42845	rno-miR-125b-2-3p	−0.670	−1.591	0.205
29575	rno-miR-32-3p	−0.688	−1.611	0.205
148594	rno-miR-466d	−0.703	−1.628	0.261
148280	rno-miR-466b-2-3p	−0.710	−1.636	0.261
148483	rno-miR-466b-1-3p	−0.756	−1.689	0.306
42770	rno-miR-665	−0.888	−1.850	0.354
17896	Rno-miR-21-3p	−1.095	−2.136	0.260

*P-values have been corrected for multiple testing by the Benjamini and Hochberg adjustment method.

### Validation of microarray data by qRT-PCR

Validation of microarray data was carried out by qRT-PCR for eight of the most strongly regulated miRNAs. **Table 2** shows that numerical data obtained from microarray analysis were in line with those from qRT-PCR analysis. Statistical analysis revealed that four miRNAs (miR-29b-3p, miR-145-5p, miR-24-2-5p, miR-665) were significantly regulated (P<0.05), three miRNAs (miR-21-3p, miR-466b-2-3p, miR-466d) tended to be significantly regulated (P<0.15), and one miRNA (miR-34a-5p) was not confirmed to be significantly regulated by qRT-PCR (**Table 2**).

**Table 2:** Validation of microarray results using qRT-PCR.

	Mean fold changes		P-value*	
miRNAs	microarray	qRT-PCR	microarray	qRT-PCR
rno-miR-34a-5p	1.459	1.362	0.036	0.350
rno-miR-29b-3p	1.452	1.572	0.020	0.015
rno-miR-145-5p	1.445	1.783	0.020	0.011
rno-miR-24-2-5p	1.438	1.518	0.027	0.049
rno-miR-21-3p	−2.136	−1.828	0.026	0.068
rno-miR-665	−1.850	−1.607	0.035	0.002
rno-miR-466b-2-3p	−1.636	−1.905	0.026	0.140
rno-miR-466d	−1.628	−2.151	0.026	0.082

*P-values of microarray data have been corrected for multiple testing by the Benjamini and Hochberg adjustment method.

### *In silico*-target prediction of differentially expressed miRNAs and functional analysis

Target prediction was performed for the 17 most strongly regulated miRNAs by using three online free available algorithms. As shown in **Table S3**, a total of 1,642 target genes were predicted for the 12 most strongly down-regulated miRNAs and 144 target genes were identified for the 5 most strongly up-regulated miRNAs. In order to validate at least some of the *in silico*-predicted target genes, we determined mRNA levels of a selected set of target genes predicted from down-regulated and up-regulated miRNAs by means of qRT-PCR. As shown in **Table 3**, the mRNA levels of 15 out of 17 target genes from down-regulated miRNAs in skeletal muscle were significantly greater in the niacin group than in the control group (P<0.05). The mRNA levels of all target genes of the up-regulated miRNAs in skeletal muscle were slightly lower in the niacin group than in the control group, even though these effects were not significant.

**Table 3:** Validation of predicted target mRNAs using qRT-PCR.

Target mRNA	Control	Niacin	P-value*
	Fold of control		
*Target mRNA of up-regulated miRNAs*			
BDNF	1.00±0.27	0.90±0.26	0.53
DUSP6	1.00±0.28	0.83±0.37	0.43
INSIG1	1.00±0.12	0.98±0.39	0.93
MRAS	1.00±0.16	0.93±0.39	0.66
SLC6A1	1.00±0.30	0.73±0.37	0.25
UBE2A	1.00±0.31	0.85±0.54	0.64
*Target mRNA of down-regulated miRNAs*			
ACSL3	1.00±0.32	2.81±2.47	0.07
ACSL4	1.00±0.53	3.03±2.30	0.04
CAV1	1.00±0.22	1.73±0.30	0.01
CD36	1.00±0.41	1.54±0.46	0.06
GHR	1.00±0.28	2.45±0.54	0.01
GK	1.00±0.54	1.62±0.37	0.03
GLUT4	1.00±0.54	3.29±0.61	0.01
GLUT8	1.00±1.05	4.24±1.43	0.01
IGF1	1.00±0.41	3.18±1.08	0.01
MAPK10	1.00±0.24	2.20±0.98	0.01
NFACT3	1.00±0.43	4.68±3.80	0.04
NFKB1	1.00±0.34	1.79±0.76	0.02
NPY1R	1.00±0.25	1.45±0.35	0.02
SDHD	1.00±0.47	2.31±0.38	0.01
SMURF2	1.00±0.22	1.47±0.49	0.02
SOD2	1.00±0.20	3.04±1.12	0.01
STAT3	1.00±0.39	2.33±0.62	0.01

Data are means ± SD, n = 6 rats/group.

*P-values according to one-way ANOVA.

To explore the biological implication of the predicted targets, we performed gene-term enrichment analysis using Gene Ontology (GO) categories (molecular function, cellular component, biological process) and Kyoto Encyclopedia of Genes and Genomes (KEGG) pathway analysis by using the DAVID Functional Annotation Chart tool.

**Figure 2:**
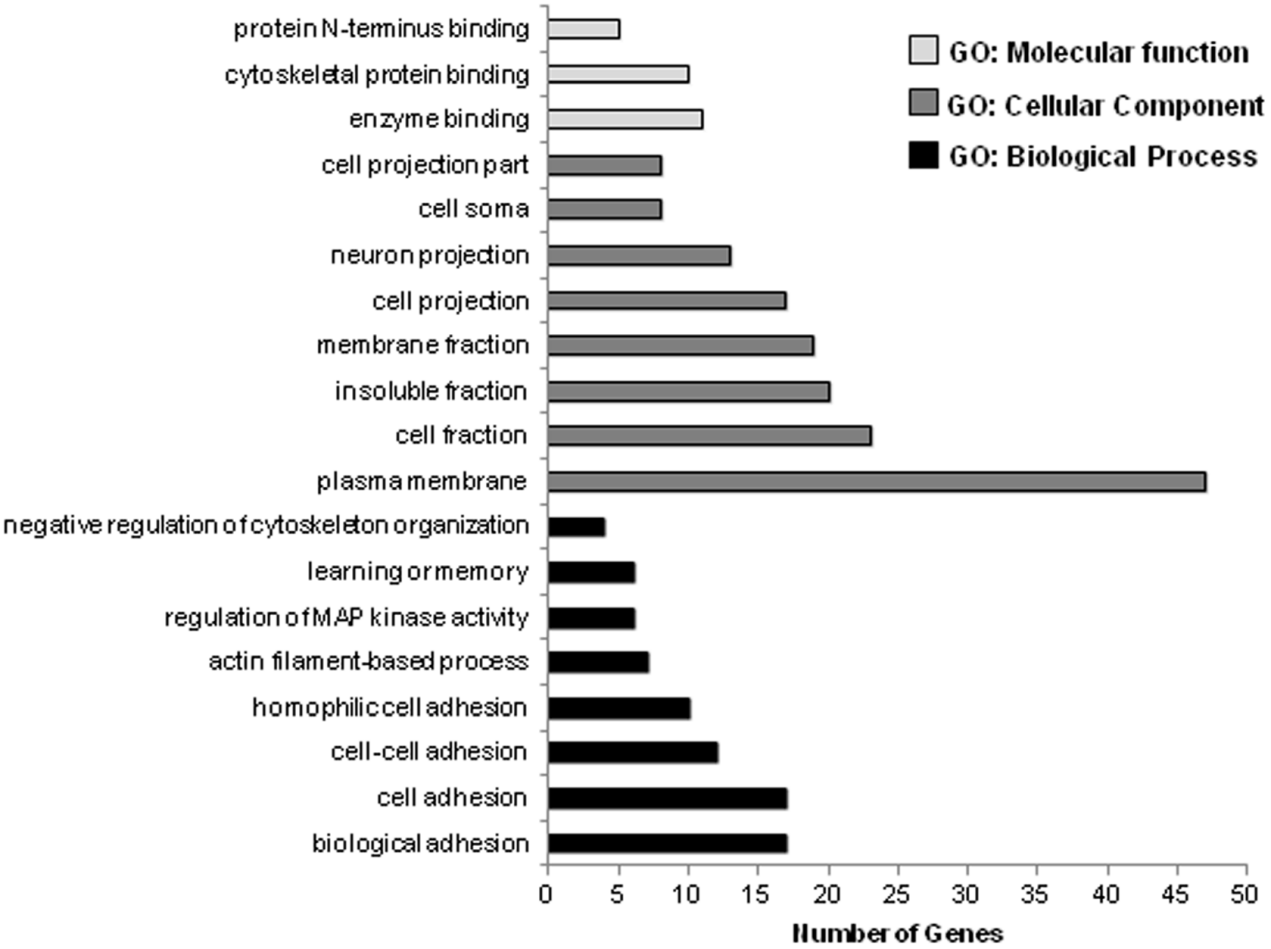
Gene ontology (GO) analysis of the target genes of the 5 up-regulated miRNAs in *M. rectus femoris* of obese Zucker rats fed either a control diet with 30 mg supplemented niacin/kg diet (control group) or a high-niacin diet with 780 mg supplemented niacin/kg diet (niacin group) for 4 wk. The GO terms were sorted by the number of genes in an ascending order from top to bottom (P-value <0.005).

Gene-term enrichment analysis of the target genes predicted from the 5 up-regulated miRNAs revealed that most genes were involved in enzyme binding, cytoskeletal protein binding, and protein N-terminus binding (GO category: molecular function; P<0.01), plasma membrane, cell fraction, membrane fraction, cell fraction, and cell projection (GO category: cellular component; P<0.01), and biological adhesion, cell adhesion, actin filament-based process, and regulation of MAP kinase activity (GO category: biological process; P<0.01; **Figure 2**).

**Figure 3:**
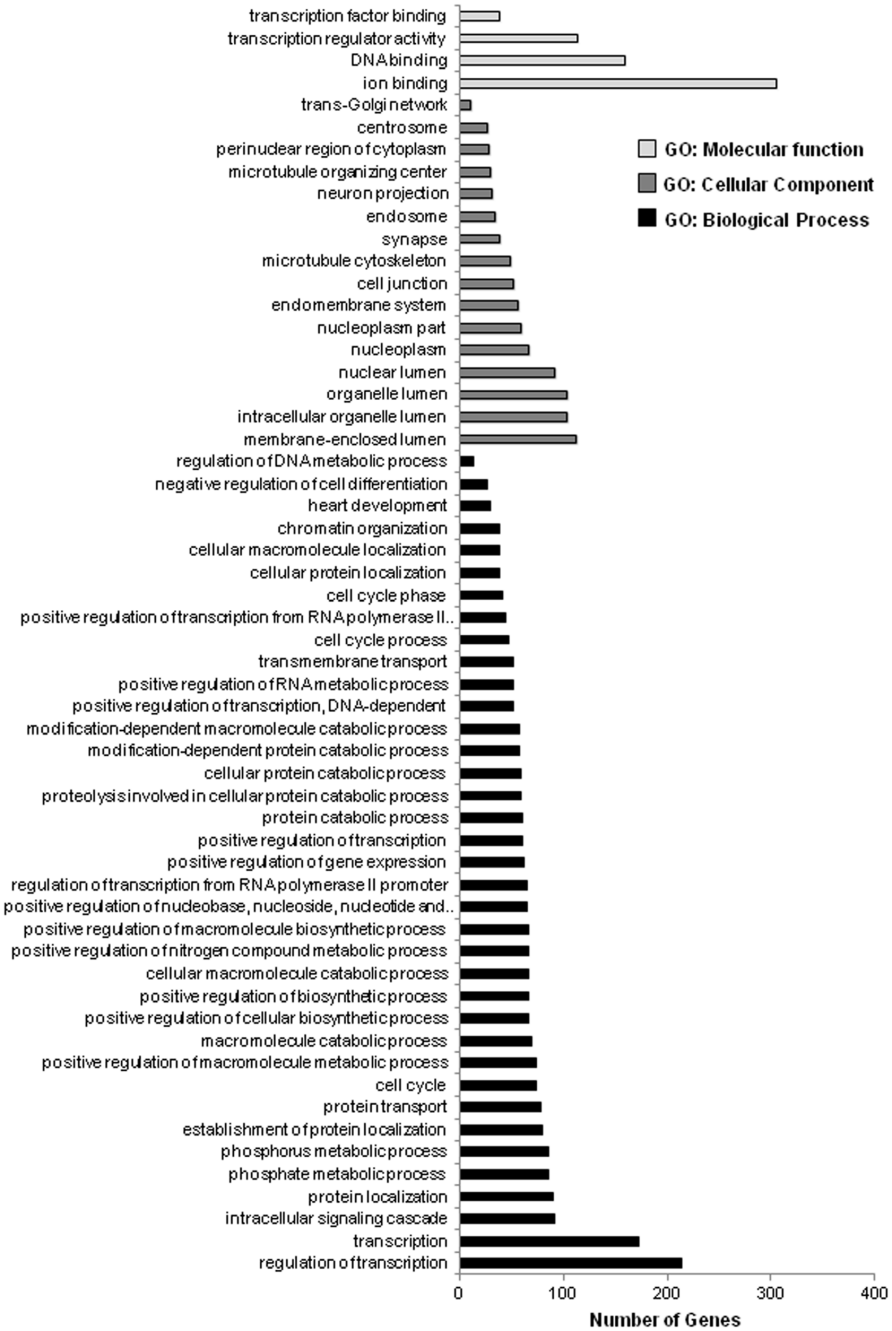
Gene ontology (GO) analysis of the target genes of the 12 down-regulated miRNAs in *M. rectus femoris* of obese Zucker rats fed either a control diet with 30 mg supplemented niacin/kg diet (control group) or a high-niacin diet with 780 mg supplemented niacin/kg diet (niacin group) for 4 wk. The GO terms were sorted by the number of genes in an ascending order from top to bottom (P-value <0.001).

For gene-term enrichment analysis of the targets predicted from the 12 down-regulated miRNAs the stringency of the filter (EASE score) was set higher (from P<0.01 to P<0.001) due to the markedly greater number of predicted targets. According to this, most target genes predicted from the 12 down-regulated miRNAs were involved in ion binding, DNA binding, transcription regulator activity, and transcription factor binding (GO category: molecular function; P<0.001; **Figure 3**), membrane-enclosed lumen, intracellular organelle lumen, organelle lumen, and nuclear lumen (GO category: cellular component; P<0.001; **Figure 3**), and regulation of transcription, transcription, intracellular signaling cascade, protein localization, regulation of transcription from RNA polymerase II, and positive regulation of gene expression (GO category: biological process; P<0.001; **Figure 3**).

To identify regulatory pathways influenced by the predicted targets from the up- and down-regulated miRNAs, gene-term enrichment analysis was performed using the KEGG pathway database. Enriched KEGG pathways (EASE score <0.05) identified from the 144 putative targets of the up-regulated miRNAs included the MAPK signaling pathway, and those from the 1,642 putative targets of the down-regulated miRNAs included neuroactive ligand-receptor interaction, ubiquitin mediated proteolysis, cell cycle, wnt signaling pathway, RNA degradation and adipocytokine signaling pathway (**Table 4**).

**Table 4:** KEGG pathway analyses of the predicted target genes of differentially expressed miRNAs with P<0.05.

Pathway	P-value*	Genes
*Putative targets of the up-regulated miRNAs*		
MAPK signaling pathway	0.025	BDNF, DUSP2, MAP2K1, MRAS, MAP3K7IP2, MAP3K12, DUSP6
*Putative targets of the down-regulated miRNAs*		
Neuroactive ligand-receptor interaction	0.017	CALCR, GABRB3, GRIK1, TRHR, F2RL1, GLRA2, GNRHR, GABBR2, NR3C1, LPAR1, HCRTR2, CNR1, P2RY1, ADRA2B, GHR, GRID1, GABRA2, GABRA1, GRIA3, NPY1R, GRIA4, GRM1, ADRB2, P2RY10, AGTR1B, SSTR1, GRM6, HTR2C
Ubiquitin mediated proteolysis	0.003	UBE2A, SYVN1, XIAP, UBE3A, UBA6, BIRC6, UBE2I, ANAPC10, HERC3, UBOX5, UBE3C, UBE2C, UBE2B, RBX1, CUL3, UBE2N, ERCC8, EDD4, SIAH1A, RHOBTB2, SMURF2, BXW11
Wnt signaling pathway	0.023	FZD8, PPP3R1, SMAD3, CXXC4, MAPK10, DAAM1, TCF7L2, PRKX, RBX1, PRKCB, SIAH1A, JUN, NFAT5, WIF1, PLCB1, FBXW11, MYC, NFATC3
Cell cycle	0.005	CDC7, YWHAZ, CDC14A, RBL2, SMAD3, ANAPC10, CHEK1, YWHAE, RBX1, CDKN1C, RAD21, HDAC2, BUB1B, CCNA2, MYC, STAG2, STAG1, SMC1B
RNA degradation	0.007	PATL1, PAPOLA, WDR61, CNOT6L, PNPT1, PAPD7, PAPOLG, XRN1, CNOT4, DDX6, C1D
Adipocytokine signaling pathway	0.014	CD36, SLC2A4, PRKAG2, NFKBIA, MAPK10, ACSL4, ACSL3, STAT3, CPT1A, CAMKK2, PCK1

*P-values have been corrected for multiple testing by the Benjamini and Hochberg adjustment method.

A further functional analysis of the predicted targets from the 17 most strongly regulated miRNAs was carried out by identifying those targets which encode transcription factors. From the approximately 1,800 predicted targets, we identified 49 mRNA targets encoding transcription factors. According to the GeneCards Human Gene Database (Weizmann Institute of Science), five out of these transcription factors, namely AFF4, BTF3L4, GTF2B, GTF2H1, GTF2H3, belonged to the class general transcription factors. The remaining transcription factors belonged to the class specific transcription factors (syn. upstream transcription factors). In **Table 5**, the main functions of these specific transcription factors provided from GeneCards are summarized.

**Table 5:** Specific transcription factors identified as predicted targets of up- and down-regulated miRNAs.

Gene symbol	Main function
*Predicted targets of up-regulated miRNAs*	
EGR2	learning; long term potentiation
ELF2	Isoform 1: synergistically with RUNX1; Isoform 2: repression of RUNX1-mediated transactivation
HBP1	cell cycle; Wnt pathway
KLF11	cell growth; induction of apoptosis
NFIA	metallothionein IIA
TFED	T-cell-dependent antibody responses; autophagy
ZFP367	Isoform 1: transcriptional activation of erythroid genes
*Predicted targets of down-regulated miRNAs*	
AHCTF1	nuclear pore complex (NPC); mitosis
ATF1	cell proliferation and transformation; repression of the expression of FTH1 and other antioxidant detoxification genes
ATF3	cellular stress response
CEBPG	binding positive regulatory element-I of the IL-4 gene
CNBP	specificity to the sterol regulatory element (SRE); sterol-mediated repression
CREM	spermatogenesis; spermatid maturation
E2F6	regulation of E2F-dependent genes whose products are required for entry into the cell cycle but not for normal cell cycle progression
E2F7	angiogenesis, DNA damage response; endocycle; placental development
FOXC1	cell viability; resistance to oxidative stress in the eye
FOXG1	brain and telencephalon development
HBP1	cell cycle; Wnt pathway
HIVEP1	T-cell activation; apoptosis
HIVEP3	immunity; inflammation; adult bone formation
HOXC4; HOXD1	part of a developmental regulatory system that provides cells with specific positional identities on the anterior-posterior axis
IHX8	neuron and mesenchymal cell differentiation
IRF1	hematopoiesis; immune responses; cell proliferation and differentiation; tumor suppression
IRF9	interferon stimulated genes
MEF2A	growth factor- and stress-induced genes; MAPK signaling
MEF2C	cardiac morphogenesis and myogenesis; vascular development
MIZF	G1/S phase transition
MTF1	metallothionein I
MTF2	embryonic stem cell self-renewal and differentiation
NKX6-1	islet beta cells; insulin
OTX2	development of brain and sense organs
POU4F1	neuronal lineages
PTF1A	formation of pancreatic acinar and ductal cells; cerebellar development
RUNX2	osteoblastic differentiation; skeletal morphogenesis
TCF7L2	Wnt signaling pathway; epithelial stem-cell compartment of the small intestine
TWIST2	proinflammatory cytokines; postnatal glycogen storage and energy metabolism
ZNF367	transcriptional activation of erythroid genes
ZFP423	BMP signaling; olfactory neurogenesis
ZNF521	BMP signaling; hematopoietic system
ZFPM2	heart morphogenesis; development of coronary vessels from epicardium

## Discussion

This is the first study reporting about the influence of pharmacological niacin doses on the miRNA expression profile in skeletal muscle of obese Zucker rats. We have recently observed in this animal model that administration of a pharmacological dose of niacin for 4 wk causes a muscle fiber shift from type II to type I and increases the percentage of type I fibers in skeletal muscle [7]. In addition, we found that the expression of genes involved in fatty acid transport, mitochondrial fatty acid import and oxidation, oxidative phosphorylation and angiogenesis and of genes encoding molecular regulators of muscle fiber distribution, like PPARδ, PGC-1α and PGC-1β, in skeletal muscle is elevated by niacin administration [7]. However, the molecular mechanisms underlying the alteration of gene expression by pharmacological niacin doses in skeletal muscle are completely unknown. Because miRNAs have been shown to play a critical role for gene expression through inducing miRNA-mRNA interactions which results in the degradation of specific mRNAs or the repression of protein translation [13], [31], we herein investigated alterations in the miRNA expression profile of skeletal muscle in response to niacin administration.

As expected, administration of the pharmacological niacin dose (0.78 g niacin/kg diet) for 4 wk to the obese Zucker rats resulted in an about 2-fold elevation of plasma NAM levels compared to the control rats which received a physiological niacin dose sufficient to cover their niacin requirement. The elevation of plasma NAM levels was in the same range as reported in other studies, in which a similar niacin dose was fed to the rats [26], [32]. In contrast, nicotinic acid and NUA could not be detected in plasma of the niacin group. This finding is also in line with another study [26], and is explained by the fact that nicotinic acid from the diet is rapidly converted to the coenzyme NAD in the intestine and liver and finally converted into NAM which is released into the blood stream [33].

The main finding of the present study is that administration of the pharmacological niacin dose for 4 wk caused a significant alteration of the miRNA expression profile in skeletal muscle of obese Zucker rats. This was evidenced by the observation that a total of 42 miRNAs were found to be differentially regulated by administration of the pharmacological niacin dose. Using a biostatistics approach, we could demonstrate that the most strongly up-regulated (log2 ratio ≥0.5) and down-regulated (log2 ratio ≤−0.5) miRNAs target 144 and 1,642, respectively, mRNAs indicating that pharmacological niacin doses can influence a large set of genes through miRNA-mRNA interactions. In an attempt to elucidate the biological implications of the niacin-induced changes in miRNA expression, we performed gene-term enrichment analysis using GO categories and KEGG pathways for the mRNAs targeted by the differentially regulated miRNAs. Following this approach we observed that many of the predicted target mRNAs from the most strongly down-regulated miRNAs were involved in DNA binding, transcription regulator activity, transcription factor binding, regulation of transcription, intracellular signaling cascade, regulation of transcription from RNA polymerase II, and positive regulation of gene expression. This indicates that molecular processes dealing with gene transcription are activated by niacin treatment because the mRNAs from the down-regulated miRNAs are less targeted and, thus, less degraded. In line with the influence of niacin on molecular processes dealing with gene transcription, we identified amongst the predicted mRNA targets approximately 50 mRNAs encoding transcription factors, which were classified according to their function into general and specific transcription factors. The main part belonged to the class specific transcription factors, which usually bind upstream of the initiation site to specific recognition sequences of the regulated gene to stimulate or repress its transcription. Several of these specific transcription factors, such as HBP1, KLF11, AHCTF1, ATF1, E2F6, E2F7, HBP1, IRF1, MEF2A, and MIZF, are known to be involved in cell cycle regulation, whereas others, like ATF3, FOXC1, HIVEP3, IRF9 and TWIST2, play a role in cellular stress response. Although it is difficult to directly relate these functions and the functions of most other specific transcription factors to the recently observed niacin-induced changes of the skeletal muscle phenotype, one exception regards E2F7, which could be identified as one of the predicted targets of the down-regulated miRNAs by niacin. While the best-known function of E2F7 and other members of the E2F family has long been their role in cell cycle regulation, a very recent study demonstrated that E2F7 promotes angiogenesis through transcriptional activation of the vascular endothelial growth factor A (VEGFA) [34]. This might provide an explanation for the recent finding that niacin treatment causes an up-regulation of VEGFA and presumably also VEGFB in skeletal muscle of rats [7] and sheep [35]. The observation that the minor part of the transcription factors targeted by the niacin-regulated miRNAs were general transcription factors, like AFF4, BTF3L4, GTF2B, GTF2H1, and GTF2H3, does not exclude an important role for the niacin-induced muscle fiber switching in obese Zucker rats [7], because general transcription factors are essential for gene transcription to occur, because they are part of the large transcription preinitiation complex that interacts with RNA polymerase directly. Muscle fiber switching is known to be initiated by up-regulation of the abovementioned key regulators of muscle fiber distribution, PGC-1α, PGC-1β, and PPARδ [8], [9], [11], [10], but it is possible that activation of general transcription factors contributes to the activation of these key regulators. In particular PGC-1α and PGC-1β regulate the muscle metabolic phenotype by activating a great number of nuclear receptors and additional transcription factors, e.g., PGCs co-activate PPARs, nuclear respiratory factor-1 (NRF-1) and estrogen-related receptor α (ERRα), all of which play central roles in regulating genes involved in fatty acid oxidation, thermogenesis, oxidative phosphorylation and mitochondrial biogenesis [11], [36]. In addition, both PGCs co-activate the myocyte enhancer factor 2 (MEF2) family of transcription factors, which stimulate specifically the expression of MHC genes from oxidative fibers [10]. These molecular effects on transcription factor activity likely explain that pharmacological niacin doses up-regulate specifically the type I fiber-specific MHCI isoform, and stimulate transcription of genes involved in mitochondrial fatty acid catabolism, citrate cycle, oxidative phosphorylation, and thermogenesis [7]. In agreement with this assumption, we found that CD36 (fatty acid translocase) and CPT1A (carnitine-palmitoyltransferase 1A), which encode genes involved in cellular and mitochondrial fatty acid uptake, respectively, were among the predicted targets of the most strongly down-regulated miRNAs, implying that CD36 and CPT1A mRNAs are less targeted by the miRNAs and less degraded. In addition, these findings could be further substantiated by qRT-PCR measurements of target mRNAs, which were performed to reinforce our observations from *in silico*-target prediction. For instance, we found that CD36, SDHD (encoding the citrate cycle enzyme succinate-dehydrogenase), but also ACSL3 and ACSL4, both of which encode acyl-CoA synthetases involved in fatty acid activation, were up-regulated in skeletal muscle of the niacin group compared to the control group. Moreover, CAV1 encoding the caveolin 1 protein, which also has been proposed to play a role in cellular fatty acid uptake [37], [38] and was shown to be coordinately expressed with CD36 in skeletal muscle [39], was also found to be up-regulated in the niacin group. Collectively, these findings are in line with our recent findings in obese Zucker rats that niacin stimulates the expression of genes involved in fatty acid uptake and utilization and citrate cycle [7]. Thus, our findings suggest that at least some of the molecular effects of niacin on skeletal muscle phenotype are mediated through miRNA-mRNA interactions. Further support for the assumption that niacin exerts some of its effects via the gene regulatory potential of miRNAs could be provided from gene-term enrichment analysis using the KEGG pathway database. Namely, we found that RNA degradation was one of the enriched KEGG pathways identified from the large set of putative targets from the down-regulated miRNAs. In addition, Wnt signaling pathway, which is critical for several features of skeletal muscle physiology including formation of muscle fibers during pre- and postnatal myogenesis was identified as an enriched signaling pathway. Wnt signaling also regulates the formation of neuromuscular synapses, by modulating the differentiation of pre- and postsynaptic components, particularly regarding the clustering of acetylcholine receptors on the muscle [40], a fact that probably explains that plasma membrane, membrane fraction, and synapse were identified as significantly enriched gene-terms associated with the mRNAs targeted by the niacin-regulated miRNAs. Moreover, we showed that the MAPK signaling pathway, which was previously reported to be regulated by niacin administration [41], is an enriched KEGG pathway identified from the putative targets of the niacin-regulated miRNAs. An influence of niacin on MAPK signaling was also evident from the observation that several MAPK isoforms including MAP2K1, MAP3K12, MAP3K7IP, MAP2K1IP1, MAP3K4, MAP4K3, MAP7D2, MAPK10, MAPK1IP1L were identified as targets of the niacin-regulated miRNAs. Since MAPK is an important kinase regulating the function of proteins via phosphorylation, it is likely that at least some of the effects of niacin in skeletal muscle are mediated at the posttranslational level. This assumption is further strengthened by the finding that CMPK1, IPMK, SGK1, AAK1, CAMKK2, DCLK1, ETNK1, GALK2, NEK1, NEK8, PANK3, and many other kinases were found to be targets of the niacin-regulated miRNAs. Our finding that MAPK signaling is one pathway affected by the putative targets of the niacin-regulated miRNAs agrees also with the view that niacin alters expression of genes involved in muscle fiber distribution through miRNA-mRNA interactions because MAPK was shown to regulate PGC-1 [42]. Furthermore, the observation that adipocytokine signaling pathway is amongst the pathways targeted by the niacin-regulated miRNAs possibly provides an explanation for recently published anti-inflammatory effects of niacin in adipocytes and macrophages [43]–[45].

It remains to be established how niacin mediates its effects on gene expression in general and miRNA expression in specific in skeletal muscle because skeletal muscle does not express the niacin receptor HCA_2_ making a direct effect of niacin unlikely. Alterations in the local microcirculatory haemodynamics caused by dilatation of skeletal muscle arterioles are known to increase capillary shear stress and, thereby, activate signaling pathways and gene expression in skeletal muscle via muscle fiber membrane-bound mechanoreceptors [46]. Although vasodilatation is also one frequent unwanted side-effect of niacin treatment [47], this effect is unlikely to contribute to the niacin-induced changes in miRNA expression in skeletal muscle, because niacin-induced vasodilatation occurs only in the dermal blood vessels and is responsible for the characteristic reddening of the skin, also called flushing, due to HCA_2_ receptor-mediated secretion of vasodilatatory prostaglandins from Langerhans cells and keratinocytes in the skin. However, it is well documented that niacin besides altering blood levels of free fatty acids induces several humoral changes, like increases in the plasma levels of epinephrine, corticosterone, glucagon, growth hormone, adiponectin and leptin [48]–[51]. All of these signals were shown to influence gene expression and cellular signaling in different tissues, and some of them were even reported to influence miRNA expression [51], [52]. Thus, future studies have to delineate which of these signals contribute to the alteration of gene expression in skeletal muscle.

It is important to note that the pharmacological niacin dose used in the present study was administered as nicotinic acid and not as NAM. In the European literature, niacin is a collective term for nicotinic acid and NAM, whereas in the US American literature niacin is exclusively used for nicotinic acid. Despite the fact that nicotinic acid and NAM can be converted into each other in the body [33], the effect of NAM on the miRNA profile may be different from that of nicotinic acid because NAM and nicotinic acid possess different pharmacokinetic properties, which amongst other are responsible for the less-pronounced adverse effects (no skin flushing) of NAM. Thus, future studies have to clarify whether nicotinic acid and NAM differ with regard to the miRNA expression profile in skeletal muscle.

In conclusion, the present study shows for the first time that pharmacological niacin doses alter the expression of miRNAs in skeletal muscle of obese Zucker rats. Results obtained from in silico-target gene prediction show that approximately 1,800 mRNAs are putative targets of niacin-regulated miRNAs. In addition, biostatistics analysis revealed that the niacin-regulated miRNAs target a large set of genes and pathways which are involved in gene regulatory activity indicating that at least some of the recently reported effects of niacin on transcriptional regulators of muscle fiber distribution and muscle phenotype are mediated through miRNA-mRNA interactions. Future experimental validations (miRNA knockdown, miRNA overexpression) for at least some of the predicted miRNA-mRNA interactions are necessary to confirm the results from biostatistics analysis.

## Supporting Information

Table S1 **Characteristics of gene-specific primers used for qRT-PCR validation of mRNA targets of differentially expressed miRNAs.**
(DOCX)

Table S2 **Differentially expressed miRNAs in** ***M. rectus femoris*** **of obese Zucker rats fed either a control diet with 30 mg supplemented niacin/kg diet (control group) or a high-niacin diet with 780 mg supplemented niacin/kg diet (niacin group) for 4 wk.** Spreadsheet contains all differentially expressed miRNAs with an adjusted P-value <0.05.
(XLSX)

Table S3 **Predicted target genes of the most differentially expressed miRNAs in** ***M. rectus femoris*** **of obese Zucker rats fed either a control diet with 30 mg supplemented niacin/kg diet (control group) or a high-niacin diet with 780 mg supplemented niacin/kg diet (niacin group) for 4 wk.** Spreadsheet contains target genes of the 5 most strongly up- and 12 most strongly down-regulated miRNAs predicted by at least one and a maximum of three online free available algorithms targetScan, miRanda and miRDB.
(DOCX)
