# Supplementary material for: Niacin in Pharmacological Doses Alters MicroRNA Expression in Skeletal Muscle of Obese Zucker Rats
Source: PLoS One. 2014 May 21;9(5):e98313. doi: 10.1371/journal.pone.0098313 (PMC4029970; doi:10.1371/journal.pone.0098313)
Supplement: Table S1 — Characteristics of gene-specific primers used for qRT-PCR validation of mRNA targets of differentially expressed miRNAs. (DOCX) [file pone.0098313.s001.docx]

**Table S1 - Characteristics of gene-specific primers used for qRT-PCR validation of mRNA targets of differentially expressed miRNAs.**

| Gene symbol | Primer sequence  (forward, reverse; from 5’ to 3’) | NCBI GeneBank | Product size (bp) |
| --- | --- | --- | --- |
| ACSL3 | GTAAAACTTGATTCCCGTTGAGA  GTGTCGCAGCCAGGATACA | NM_057107.1 | 307 bp |
| ACSL4 | CACCTTCGATCCCAGGAGATT  TTTGCCATAGCGTTTTTCTTAGAT | NM_053623.1 | 72 bp |
| BDNF | TGCTCTTTCTGCTGGAGGAA  TTTCTCCAGGACTGTGACCG | XM_006234684.1 | 191 bp |
| CANX | CCAGATGCAGATCTGAAGAC  CTGGGTCCTCAATTTCACGT | NM_172008 | 175 bp |
| CAV1 | CTCTTCTTCCCACCGCTGTT  TGAAGCTGGCCTTCCAGATG | NM_133651.2 | 294 bp |
| CD36 | TCGTATGGTGTGCTGGACAT  GGCCCAGGAGCTTTATTTTC | NM_031561.2 | 358 bp |
| DUSP2 | TGTGAGTGTGTGGGCATCTC  AAAGTGCTGTTTCCAACGCC | NM_001012089.1 | 130 bp |
| DUSP6 | CCCCAATCTGCCCAATCTGT  CACCACAGTTTTTGCCTCGG | NM_053883.2 | 146 bp |
| GHR | CTCGGAGTTTGCTCAGCCGCA  GGCCGCAGCGATGCACTTTT | NM_017094.1 | 415 bp |
| GK | TCTCCTGAAAGTGGTATCCCATA  GGCATAGGTCTGCTTGGAGG | NM_024381.2 | 176 bp |
| GLUT4 | GAGTTATGTGTCCATCGTGG  CGCAACATACTGGAAACCCA | NM_012751.1 | 187 bp |
| GLUT8 | GACTGCTCGGCTCCTGTGTGC  CATGGCCTCCTGGTACTGGTGTT | NM_053494.2 | 194 bp |
| IGF1 | CCCGGGACGTACCAAAATGAGCG  ATGTCAGTGTGGCGCTGGGC | NM_178866.4 | 354 bp |
| INSIG1 | GCAGCAGTAAGTTGGAGGAA  CTGGAGTGACAGAGACACAA | NM_022392.1 | 309 bp |
| MAPK10 | GTTTGGTACGACCCTGCTGA  GAGGGCTGGCCTTTGACTAC | NM_001270556.1 | 164 bp |
| MRAS | CATGCGGGAGCAATACATGC  TGGCAGTTCACGGTTGCTAT | NM_012981.2 | 438 bp |
| NFACT3 | CCAGCCATCAGGTTCAACAGA  TCCAACTTCTGCGACAGATTC | NM_001108447.1 | 294 bp |
| NFKB1 | CTTCAACATGGCAGACGACG  AACCACCATGTCCTTGGGTC | NM_001276711.1 | 403 bp |
| NPY1R | GTGGCTTCTTCTCTGCCCTT  GTTGACGCAGGTGGAGATCA | NM_001113357.1 | 450 bp |
| RPL13 | CTTAAATTGGCCACGCAGCT  CTTCTCAACGTCTTGCTCTG | XR_086310 | 198 bp |
| SDHD | TTGCTCTTGGGCCTGATTCC  GCAGATGCCTACATCGTGGT | NM_198788.2 | 228 bp |
| SLC6A1 | GGTTCCCTCCATCCTACCCT  AGAGTCTCCACTGAGCAGGT | NM_024371.2 | 535 bp |
| SMURF2 | TTTGCTGTGGAGTGATGAGC  CCACGTGTTCAATCCAACTG | NM_001107061.1 | 424 bp |
| SOD2 | CGGGGGCCATATCAATCACA  GCCTCCAGCAACTCTCCTTT | NM_017051.2 | 84 bp |
| STAT3 | ATCCTAAGCACAAAGCCCCC  GTAAGGGGCAGCACTACCTG | NM_012747.2 | 327 bp |
| TOP1 | GAAGAACGCTATCCAGAAGG  GCTTTGGGACTCAGCTTCAT | NM_022615 | 137 bp |
| TRIM63 | AAGGCAGCCACCCGATGTGC  GCCTGGTGAGCCCCGAACAC | NM_080903.1 | 112 bp |
| UBE2A | TGTGGAAACCACAGGACAACT  CAGTCACGCCAGCTTTGTTC | NM_001013933.1 | 327 bp |
